# A semi-solid in vitro biofilm model for evaluating antimicrobial potency and biofilm-specific activity

**DOI:** 10.1016/j.bioflm.2025.100328

**Published:** 2025-11-05

**Authors:** Albert Fuglsang-Madsen, Lasse Andersson Kvich, Nicole Lind Henriksen, Rasmus Kristensen, Jonas Rosager Henriksen, Anders Elias Hansen, Thomas Bjarnsholt, Tim Holm Jakobsen

**Affiliations:** a Department of Health Technology, The Technical University of Denmark, Kongens Lyngby, Denmark; b Department of Surgery, Center for Surgical Science, Zealand University Hospital, Køge, Denmark; c Department of Veterinary- and Animal Sciences, University of Copenhagen, Frederiksberg, Denmark; d Costerton Biofilm Centre, Department of Immunology and Microbiology, University of Copenhagen, Copenhagen, Denmark; e Department of Clinical Microbiology, Rigshospitalet, Copenhagen, Denmark

**Keywords:** Biofilm *in vitro* models, *Staphylococcus*, Biofilm, Antimicrobials, Antimicrobial screening, Infection microbiology

## Abstract

Biofilms play a critical role in chronic bacterial infections, and new potent antimicrobials are urgently needed to address the escalating problem of antimicrobial resistance to existing therapies. To support the development of such therapeutics, there is a pressing need for biofilm models that better recapitulate the microenvironment of *in vivo* conditions. Existing *in vitro* assays, such as the widely used minimum biofilm eradication concentration (MBEC) assay, rely on liquid cultures that poorly reflect the structural and physiological characteristics of tissue-associated biofilms.

To address these limitations, we developed the Modified Crone's Model (MCM), a reproducible, semi-solid biofilm model that embeds bacteria in soft-tissue-like agar-based matrices. We established the MCM as a platform for evaluating the antimicrobial and specific anti-biofilm activity of novel and existing compounds. Biofilms grown in the MCM displayed consistent growth, *in vivo*-like morphology, and reduced variability compared to liquid-culture systems. Notably, antimicrobial susceptibility rankings in the MCM differed substantially from traditional assays, emphasizing that model-specific conditions can markedly affect the evaluation of antimicrobial potency and should be considered when selecting biofilm test systems.

Using the MCM, we screened a panel of therapeutic agents and identified two unsaturated fatty acids – *cis*-2-decenoic acid and *cis*-11-methyl-2-dodecenoic acid – as potent antibiotic potentiators with intrinsic anti-biofilm activity, undetectable in microbroth dilution assays. We further demonstrated the MCM's adaptability by replicating susceptibility profiles in biofilms grown on porcine bone tissue and implant surfaces, with no significant differences from agar-based biofilms.

The MCM offers a simple and reproducible platform for preclinical antimicrobial screening under semi-solid growth conditions that better reflect the spatial and diffusional constraints of biofilm-associated infections.

## 1 Introduction

Accurate *in vitro* models are essential for advancing drug development, particularly in the field of infectious diseases. Preclinical models that fail to replicate the complex biological conditions of host environments risk producing misleading results and contribute to the poor translational success of many therapeutic candidates. This is especially true in the study of bacterial infections, where the physiological state of bacteria can dramatically influence antibiotic susceptibility [[1], [2], [3], [4]].

Bacterial biofilms are structured communities that contain dormant and slow-growing cells which are particularly tolerant to antibiotics. Antibiotic susceptibility varies significantly between these states, with biofilm-associated bacteria exhibiting up to 1000-fold increase in tolerance compared to their planktonic counterparts, depending on the antibiotic type [[5], [6], [7], [8], [9]], environmental conditions, and culturing media [3,10,11].

To address this challenge, developing new therapeutic strategies and compounds capable of eradicating biofilm-associated bacteria and overcoming the protective barriers that limit antibiotic efficacy is essential [12]. Developing novel antimicrobial agents, particularly those effective against biofilm-associated bacterial communities, requires reliable preclinical models that accurately mimic *in vivo* conditions. Traditional *in vitro* biofilm models often rely on liquid-phase culturing systems, which fail to replicate the host tissues' spatial structure, physical constraints, and diffusion limitations [11,[13], [14], [15], [16]]. These simplified systems may overlook critical aspects of biofilm physiology that influence the efficacy of antimicrobial molecules, leading to poor translation of laboratory findings.

Numerous environmental parameters, including oxygen and nutrient gradients, mechanical forces, and the structural characteristics of the surrounding matrix, influence biofilm formation. These conditions are rarely reproduced in conventional assays. In particular, the lack of spatial confinement and semi-solid environments in liquid-phase models limits their ability to capture key features of soft-tissue infections, bone-embedded biofilms, or biofilm growth on implanted materials [2,17].

Although complex biofilm models simulate disease-relevant conditions better, they often require specialized equipment, custom molds, or complex media, limiting reproducibility and scalability [1,2,10,17,18]. There remains a need for a simple, accessible, and physiologically relevant biofilm model that can support reproducible biofilm growth and be broadly adopted for antimicrobial screening.

In this study, we present and characterize the Modified Crone's Model (MCM), a semi-solid, agar-based biofilm model, designed to provide a more representative platform for evaluating the antimicrobial activity of therapeutic compounds against biofilm-associated bacteria. Using *Staphylococcus aureus* as a model organism, we compared antimicrobial susceptibility patterns between biofilms grown in the MCM and conventional liquid-based systems. The results highlight the importance of context-specific models in preclinical drug evaluation and demonstrate the utility of the MCM in screening antimicrobial agents and adjuvants for biofilm-targeted therapies.

## 2 Materials & methods

### 2.1 Bacterial strain, antibiotics, and materials

All experiments were conducted with *S. aureus* strain S54F9 [[19], [20], [21]]. Applied antibiotics were purchased from Sigma-Aldrich (unless stated otherwise) and include levofloxacin, linezolid, rifampicin, gentamicin sulphate (TCI Europe), clindamycin hydrochloride monohydrate (TCI Europe), *cis*-11-methyl-2-dodecenoic acid a.k.a. diffusible signal factor (DSF, Chemodex Ltd.), and *cis*-2-decenoic acid (Santa Cruz biotechnology). Lysogeny broth (LB), brain heart infusion broth (BHI), and agar were utilized for bacterial culturing.

### 2.2 Construction of the biofilm model

The new *in vitro* model presented here is a modified and enlarged version of the *in vitro* model described by Crone et al. in 2015 [22], which will be referred to as the Modified Crone's Model (MCM) in this study. The rationale behind omitting e.g., host factors in the growth media of this model is to obtain more simplicity and reproducibility whilst retaining biofilm in a semi-solid environment. The reason for choosing a semi-solid rather than liquid culturing system is the hypothesis that the most crucial factor to mimic is the resulting biofilm size, as it largely affects the physical dimensions that may govern the establishment of heterogenous subpopulations with differing growth rates (due to nutrient distribution), and which alters the penetration of antimicrobials. Furthermore, embedded biofilms have a lower exchange rate of nutrients and oxygen with their surroundings, which may mimic *in vivo* conditions. In addition to physical restriction, these factors may be what confers the smaller aggregate sizes.

The MCM is constructed by letting a layer of 0.5 % agar LB broth solidify in a test tube, followed by addition of 20 μl of 0.1 OD_600_ diluted overnight culture, letting it dry, and then adding another layer of 0.5 % agar LB at lukewarm temperature to avoid heat-killing the bacteria. The two agar layers fused without delamination, forming a continuous matrix around the inoculated bacteria. After the second layer solidifies, liquid LB is added on top, and the test tubes are incubated for 96 h at 37 °C and 0 rpm. At this point, the bacterial-plane will be visible as a disc in the middle of the agar plug. Then, the drug-of-interest is added into the liquid LB *without replenishing* the LB, followed by another 72 h of incubation. Lastly, the tubes' entire contents are homogenized by mechanical disruption and plated for CFU counting. All calculations of CFU and log(CFU) were conducted as described in Supplementary Equation (1), and a detailed step-by-step protocol can be found in Supplementary Methods.

While mechanical disruption during homogenization could theoretically impact bacterial viability, this effect was not evident in our study: untreated control samples subjected to the same homogenization protocol displayed low CFU variability (e.g., Fig. 3a), suggesting that viability was preserved under these conditions. Furthermore, all experimental groups underwent identical processing, ensuring comparability across treatments. To mitigate potential heating and nutrient release during shaking, 4 °C PBS was added prior to homogenization (see Supplementary Methods for details and rationales behind experimental conditions).

### 2.3 Preparation of biofilms for confocal laser scanning microscopy

To visualize and compare biofilms grown in liquid-phase, embedded in agar, or grown *in vivo*, confocal laser scanning microscopy (CLSM) was conducted. For liquid-phase-cultured biofilm, citric acid-buffered whole horse blood (The Danish National Serum Institute) was spun down at 2000×*g* for 15 min/5 °C to obtain plasma. Horse plasma was mixed 50:50 with BHI (SigmaAldrich) (solution A). To Ibidi μDish chambers, 1 mL of solution A was added and incubated at 37 °C/15 min/110 rpm. Then, solution A was removed and 800 μL of *S. aureus* overnight culture in LB + 5 % v/v horse plasma (OD_600_ = 0.5) was added and incubated at 37 °C/30 min/110 rpm. The media (with non-adhered planktonic cells) was carefully removed, and 4 mL liquid LB media was added followed by incubation at 37 °C/110 rpm/7 days. This protocol was a modified version of the methodology from a study by Jørgensen et al. with pre-conditioning of microtitre plates for induction of *in vitro S. aureus* biofilms [23].

For agar-phase-cultured biofilm, 1 mL 0.5 %-agar LB media was added to Ibidi μDish chambers and allowed to solidify. Then, 800 μL *S. aureus* overnight culture (OD_600_ = 0.5) was added, followed by another 2 x 1 mL 0.5 %-agar LB, which was allowed to solidify between each addition. Then, 1 mL liquid LB media was added on top, and the μDish chambers were incubated at 37 °C/0 rpm/7 days.

### 2.4 Biofilm staining for confocal laser scanning microscopy

Biofilms were stained by Syto9. For the liquid-phase cultured biofilms, 2.9 mL of the media in the μDishes were removed and 100 μL 50 μM Syto9 was added (c_final_ = 5 μM). The μDishes were swirled to mix and incubated at 37 °C/30 min before imaging by CLSM. CLSM images were obtained from live samples, real-time. For the agar-phase-cultured biofilm, the 1 mL liquid LB media was removed, and the agar surface was washed with 1 mL saline to remove planktonic cells. A 1000 μL pipette tip was used to dig a channel through the centre of the agar plug to facilitate faster diffusion of the Syto9 dye through the agar. Then, 600 μL saline and 400 μL 50 μM Syto9 was added (c_final_ = 5 μM) and incubated at 37 °C/3 h before CLSM imaging.

CLSM imaging was conducted on a Nikon Ti2 inverted microscope, equipped with a Yokogawa CSU-W1 module and a Photometrics Prime 95B sCMOS camera. Syto9 was excited at 488 nm and detected by a 520/28 BrightLine HC bandpass filter. Images were captured using a set of 4x/0.2 and 20x/0.75 CFI Plan Apochromat Lambda objectives.

### 2.5 Image analysis and 3D biofilm quantification

**Fig. 1:**
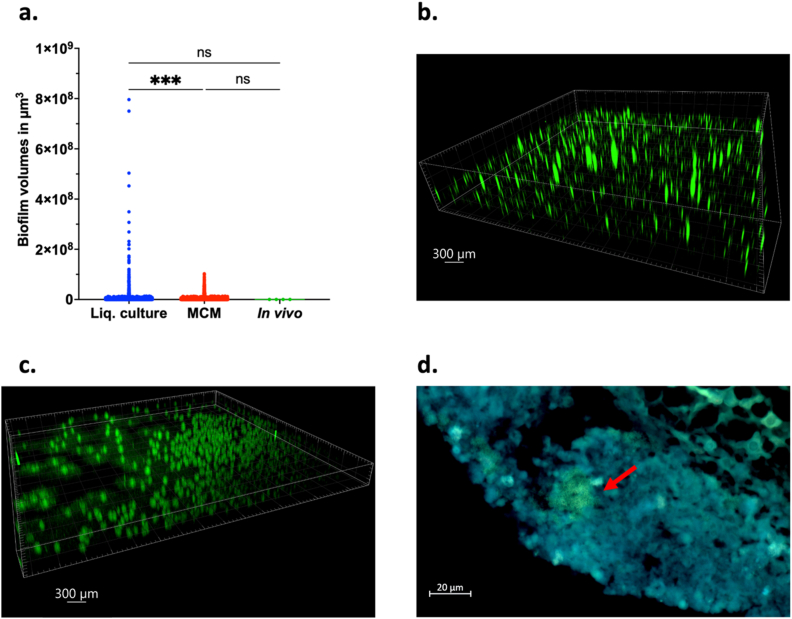
**Analyses of biofilm characteristics of *S. aureus* S54F9 in different environments**. a) Average volumes of biofilm (in μm^3^), assuming that the diameter of *in vivo* biofilms can be estimated as an average of their measured width/length. b) **Live-imaged** biofilms of *S. aureus* S54F9 grown in *liquid* culture media, after 7 days of culture, by CLSM. c) **Live-imaged***S. aureus* S54F9 biofilm aggregates in the MCM, after 7 days of culture by CLSM. d) Epifluorescence microscopy image from a section of debrided bone tissue from a pig infected with *S. aureus* S54F9, 7 days post-infection (not live-imaging). The red arrow points at the *S. aureus* biofilm aggregate. For detailed experimental information, see Supplementary Methods. The number of observations (n) were 3665 for liquid-cultured biofilms, 4812 for agar-embedded biofilms, and 4 for *in vivo* biofilms. (For interpretation of the references to colour in this figure legend, the reader is referred to the Web version of this article.)

Images were sectioned and edited for brightness and contrast in Fiji (ImageJ) and 3D projections were made from the Z-stacks for Fig. 1b–d. To quantify biofilm volumes from the live-imaged samples, Z-stacks were analysed in Fiji's 3D Object Counting tool. Five Z-stacks obtained across five different Ibidi μDishes from the liquid-cultured, in two experimental replicas (and thus two individual observation days) was included for image analysis, which yielded a total of 3665 observations. An observation was defined as a 3D object above a volume of 300 μm^3^, which would indicate aggregates of ∼100 *S. aureus* CFUs (according to the volume of 1 *S. aureus* CFU as determined in Levin & Angert 2015 [24]). Conversely, agar-embedded biofilms were obtained from four Z-stacks acquired across four different Ibidi μDishes in two experimental replicas (on the same two individual observation days as for liquid-cultured biofilms), yielding a total of 4812 observations.

### 2.6 Immunohistochemistry and fluorescent *in situ* hybridization

**Fig. 2:**
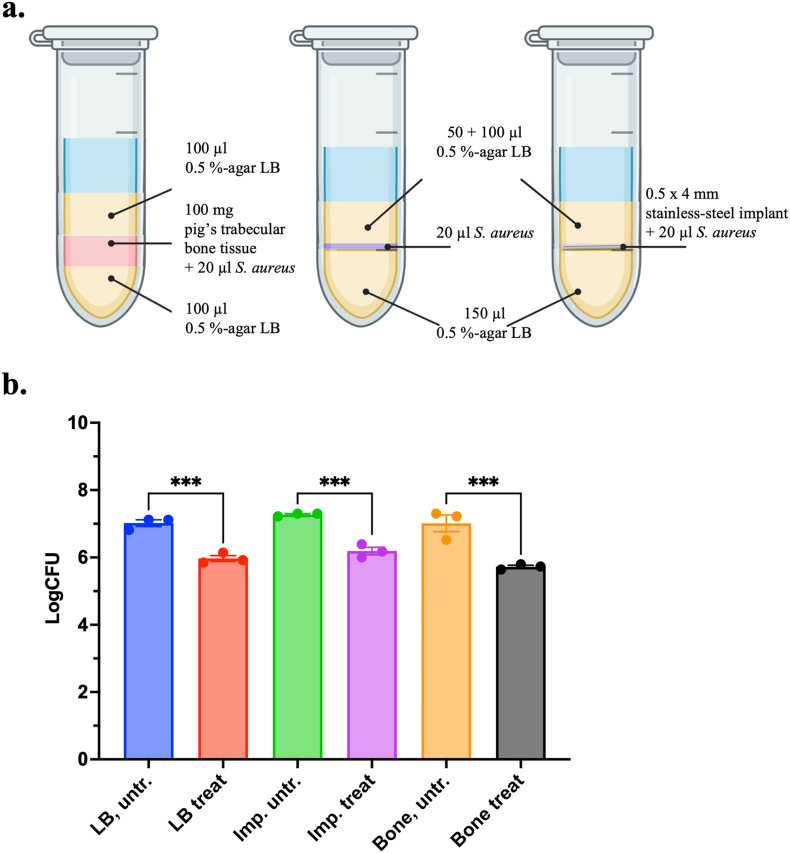
**Investigation on the effect of bone tissue- or implant-surface-associated growth environments on the levofloxacin susceptibility of *S. aureus* strain S54F9 biofilm**. a) *S. aureus* biofilms were cultured under different conditions in the MCM. In one variant, a 0.5 × 4 mm stainless-steel wire was placed in the middle of the agar plug and bacteria were inoculated onto it (righthand side). In another variant, 100 mg debrided trabecular bone tissue from a pig was placed in the middle of the agar plug, and bacteria were inoculated into it (lefthand side). b) There was no significant difference between bacterial numbers in untreated controls (across the model variants) nor between the reduction in bacterial numbers mediated by levofloxacin. However, there were significant reductions in the number of CFUs after treatment in each model variant, respectively. The number of biological replicas were 3 for each group (treated or untreated).

Paraffin-embedded *S. aureus* S54F9-infected trabecular bone tissue samples from a porcine implant-associated osteomyelitis model after 7 days of infection were used [25]. Two 4–5 μm tissue sections were cut and stained using either a combined alcian-blue and immunohistochemical (IHC) protocol for detecting *S. aureus* biofilm, as previously described [21] or fluorescent in-situ hybridization (FISH) with a CY3-conjugated oligonucleotide probe (EUB 338, 5′-3’:GCTGCCTCCCGTAGGAGT, Eurofins) targeting domain bacteria. The FISH shandon rack protocol was modified from Jensen et al. 2016 [26] by pretreatment with 0.5 mg/ml proteinase K for 10 min at 37 °C (SigmaAldrich) and hybridization was conducted at 45 °C followed by tissue dehydration with graded concentrations of alcohol and counterstaining with 3 μM DAPI (Thermo Fisher Scientific) for 15 min. IHC and FISH stained sections were visualized and imaged with light- (Olympus BX60) and fluorescence microscopy (Zeiss Axio Imager M1), respectively. Cy3, FITC and DAPI were excited by 538–562, 455–495 and 335–383 nm, respectively, and emissions were detected by 570–640, 505–555 and 420–470 filters. The exposure times were 31, 72.2 and 20.8 ms, respectively, and depth of focus 1.01, 0.93 and 0.84 μm. Only four *S. aureus* aggregates were identified from the debrided tissue samples and were included for quantitative comparisons in Fig. 2a.

### 2.7 Testing of experimental parameters of the Modified Crone's model

Throughout this work, variations in the MCM relating to biofilm maturation age and incubation with potential antimicrobial compounds were conducted, applying the MCM. For variation in biofilm maturation, biofilm cultures were matured for 1-, 4-, 7-, or 14 days, prior to 3 days of incubation with clindamycin. For variation in drug incubation time, samples were incubated with clindamycin for 1-, 3-, 7-, or 14 days, following 4 days of biofilm maturation.

Variations of the MCM with different culturing conditions were also tested. In one model variant, a 0.5 × 4 mm stainless steel wire was placed in the agar plug, onto which *S. aureus* was inoculated. In another model variant, 100 mg of debrided trabecular bone tissue from a healthy pig was placed in the middle of the agar plug, onto which *S. aureus* was inoculated (Fig. 2a).

### 2.8 Statistical data analyses

**Fig. 3:**
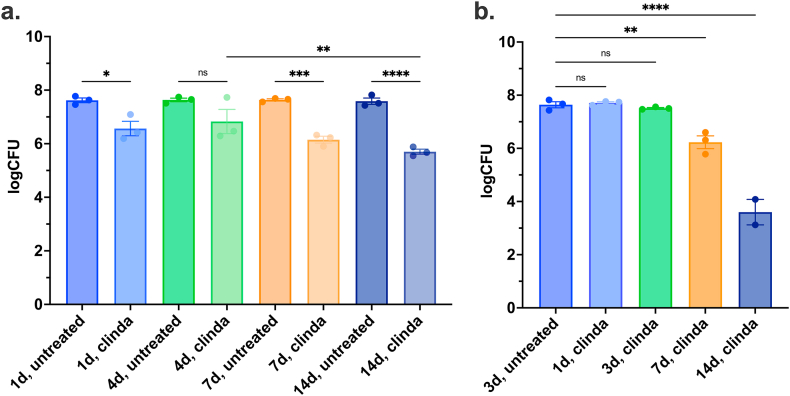
**The effect of biofilm age or exposure time to antibiotics.** a) Variation in biofilm maturation age. Total logCFU after 3 days/72hrs of treatment/exposure with or without clindamycin in *in vitro* MCM biofilms matured for 1, 4, 7, or 14 days prior to treatment. b) The effect of varying exposure/treatment time with clindamycin. Total logCFU in four-day-*in-vitro-*matured MCM *S. aureus* biofilm incubated for 3 days without treatment or for 1, 3, 7, or 14 days with clindamycin. The number of biological replicas were 3 for each group (treated or untreated).

**Fig. 4:**
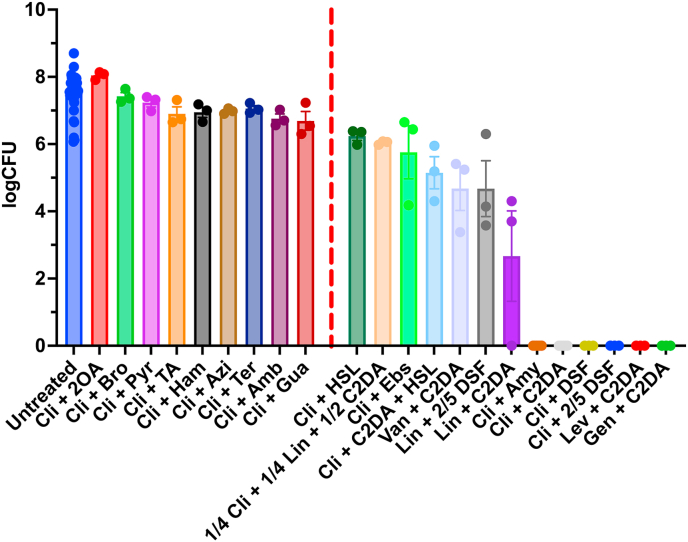
**The antimicrobial adjuvant effect of various compounds in conjunction with clindamycin compared to untreated control using the MCM.** Tested adjuvant compounds with a non-significant effect compared to the untreated control (first column) are shown to the left of the striped red line. Tested adjuvant compounds with a statistically significant reducing effect on bacterial numbers, compared to the untreated control, defined as a p-value at or below 0.05, are placed to the right of the striped red line. 2OA = 2-octenoic acid, Bro = bromhexine. Pyr = pyruvate. Azi = azithromycin. Ham = hamamelitannin. TA = tannic acid. Amb = ambroxol. Gua = guaifenesin. HSL = 3-oxo-dodecanoyl l-homoserine lactone. Ebs = ebselen. C2DA = *cis*-2-decenoic acid. DSF = diffusible signal factor, aka *cis*-11-methyl-2-dodecenoic acid. Amy = amylmetacresol. Cli = clindamycin. Van = vancomycin. Lin = linezolid. Lev = levofloxacin. The number of biological replicas were 3 for each group treatment group, while the untreated groups across all experiments are shown, yielding a total number of untreated samples, n = 51 (3 per experiment, and 17 experiments were conducted in total, with different treatment groups in each).

Tukey's tests were applied to correct multiple comparisons between biofilm volumes (Fig. 1a) and to analyse differences between levofloxacin susceptibility in MCM variants (Fig. 2b). Dunnett's tests were applied for multiple comparisons to analyse differences between various compounds in the MCM (Fig. 3b and 4 and Supplementary Figures 3 & 6). Lastly, for analysing whether there was an effect of increasing the biofilm age prior to drug incubation (Fig. 3a), Šídák's test was applied to correct multiple comparisons. All statistical analyses and mathematical model fittings were performed in GraphPad Prism version 9.4.1.

## 3 Results

### 3.1 Biofilm sizes are similar between the modified Crone's model and *in vivo* model

To assess whether biofilm aggregate sizes resembled those found *in vivo*, microscopy was conducted to visualize and compare biofilms from tissue infections with those grown in liquid cultures and agar-embedded environments. Biofilm aggregate sizes after 7 days of maturation differed, depending on the environment in which it was cultured (Fig. 1). The sizes ranged from approximately 306 μm^3^ to 0.795 mm^3^ in the microtiter plate liquid-cultured biofilms to 1129 μm^3^ to 0.103 mm^3^ in the MCM (representative images in Fig. 1b–c). For *in vivo S. aureus* S54F9 biofilm aggregates, from paraffin-embedded trabecular bone tissue samples in a porcine implant-associated osteomyelitis model (Fig. 1d), it was necessary to estimate one dimension of the biofilms, due to limitations in visualizing biofilms in tissue. Sample preparation for epifluorescence, described in the Methods, necessitates the need to cut 4–5 μm tissue sections, limiting one measurable dimension of the biofilms. Assuming the diameter of *in vivo* biofilms can be estimated as an average of their width and length, volumes were calculated and compared across the three models (Fig. 1a). The liquid-cultured biofilm volumes had a higher variance than the agar-embedded or *in vivo*-derived biofilms and were statistically significantly larger, which was also the case for *Pseudomonas aeruginosa*, a species of Gram-negative bacteria, in the MCM (Supplementary Fig. 2). In contrast, there was no statistical difference between the volumes of agar-embedded and *in vivo*-derived biofilms (Fig. 1a). Thus, since biofilm aggregate sizes in the MCM were comparable to those observed *in vivo*, but significantly different from liquid-grown aggregates, the MCM is deemed more likely to promote *in vivo*-like biofilm architecture than conventional liquid-culture models.

### 3.2 Implant-associated biofilm show similar susceptiblity to levofloxacin as agar-embedded biofilm

A stainless-steel implant or trabecular bone tissue from a pig's femur was introduced into the bacterial growth zone of the MCM (illustration, Fig. 2a) and compared to the standard MCM. When using levofloxacin as an antimicrobial agent, no differences were observed if bacteria were allowed to establish a biofilm on an implant surface (embedded in agar), in 0.5 % agar alone, or in trabecular bone tissue (embedded in agar) (Fig. 2a), when using levofloxacin as an antimicrobial agent. As there were no significant differences in levofloxacin susceptibility between biofilms formed on stainless-steel implants, in debrided trabecular bone tissue, or in LB-agar alone, the standard agar-based MCM variant was selected for subsequent experiments. This choice reflects its ease of use, broad accessibility and reproducibility.

### 3.3 Mature biofilms show increased susceptibility to clindamycin, and prolonged antimicrobial incubation time leads to increased bacterial death

Firstly, it was confirmed that antibiotics diffuse throughout – and reach equilibrium – in the MCM, during the time of drug incubation (Supplementary Fig. 3). The time-to-equilibrium at the bacterial disc was estimated to take approximately 14 h when injecting clindamycin as a proxy for antibiotics in the MCM (Supplementary Tables 1 and 2, Supplementary Fig. 3, Supplementary Equation (3), Supplementary Calculation 1, and Supplementary Methods). Varying the biofilm maturity age (Fig. 3a) while keeping the exposure/treatment time with antibiotics constant (3 days/72 h) showed that there were no differences between CFU numbers of untreated samples, despite of biofilm maturation age (non-significant comparisons are not shown). Surprisingly, the antimicrobial effect of clindamycin was more pronounced when treating older biofilms; for instance, there was a significant difference between treatment of 4-day-old vs. 14-day-old biofilms. Conversely, keeping the biofilm maturation age constant (4 days/96 h) and varying the incubation time with antimicrobials in the MCM (Fig. 3b), significantly decreased CFU numbers post-treatment aftert 7- or 14 days of exposure/treatment time, but there was no difference when the exposure time was 1- or 3 days (Fig. 3b).

### 3.4 Ranking the antimicrobial potency leads to a different hierarchy, whether determined by MIC, MBEC assays, or the Modified Crone's model

Six antimicrobials were tested against a 4-day-*in-vitro-*matured biofilm in the MCM (Supplementary Fig. 4), minimum biofilm eradication concentration (MBEC, Supplementary Fig. 5), and minimum inhibitory concentration MIC (Supplementary Fig. 6) assays, using the same *S. aureus* strain (S54F9). Based on their ability to reduce bacterial numbers, they were ranked according to their potency (Table 1).

**Table 1:** Ranking of antimicrobial potency by antimicrobial assay.

Antimicrobial assay	Ranking of antimicrobial potency
MIC	Gentamicin > Rifampicin > Levofloxacin > Clindamycin > Linezolid > DSF
MBEC	Rifampicin > DSF > Levofloxacin > Gentamicin > Clindamycin > Linezolid
Modified Crone's Model	Gentamicin > DSF > Rifampicin > Levofloxacin > Clindamycin > Linezolid

MIC = minimum inhibitory concentration. MBEC = minimum biofilm eradicating concentration. DSF = Diffusible signal factor (*cis*-11-methyl-2-dodecenoic acid).

Ranking the tested antimicrobials in the MCM and comparing to MBEC values, it was evident that the two biofilm models do not rank antimicrobials identically, despite the only difference being whether biofilms were cultured in liquid LB or embedded in LB-agar. Furthermore, it was observed that the potent anti-biofilm compound, DSF [[27], [28], [29], [30]], had no effect in the MIC tests (Supplementary Fig. 4). This is in contrast to previous studies, reporting that DSF solely exerts an effect as a signaling molecule with no intrinsic antimicrobial activity. However, it was observed reproducibly in this study.

For comparison, EUCAST clinical breakpoints, determined for *S. aureus* strain ATCC 29213, are listed in Table 2. These breakpoints can serve as an indirect measure of antibiotic potency, as a lower resistance breakpoint concentration may indicate a more potent antibiotic.

**Table 2:** Ranking of indirect antimicrobial potency from EUCAST-determined clinical breakpoints of resistance.

	Ranking of indirect antimicrobial potency
	Rifampicin > Clindamycin > Levofloxacin > Gentamicin > Linezolid

Comparing the MCM and MIC results to EUCAST breakpoints, both models misranked clindamycin and gentamicin, but the MCM more closely reflected EUCAST by correctly reproducing the relative positions of rifampicin, levofloxacin, and linezolid, whereas MIC only captured levofloxacin and linezolid accurately. However, as EUCAST breakpoints are derived from liquid-culture assays using a different *S. aureus* strain, these comparisons should be interpreted with caution and not considered directly equivalent.

Levofloxacin + linezolid, levofloxacin + rifampicin, levofloxacin + colistin, levofloxacin + clindamycin, clindamycin + linezolid, and clindamycin + rifampicin were also tested in combinations (Supplementary Fig. 7). Here, we observed that levofloxacin + clindamycin had a significantly lower antimicrobial effect than levofloxacin alone, demonstrating an antagonistic relation (Supplementary Fig. 4). It was also found that rifampicin + levofloxacin could eradicate the approximately 10^8^ CFUs after 3 days of incubation with the antimicrobials (Supplementary Fig. 7). These results demonstrate that antimicrobial potency rankings differ between the MCM and conventional MBEC or MIC assays, with the MCM identifying different top-performing treatments. While these findings suggest that the MCM may influence treatment selection differently, determining which model best predicts *in vivo* efficacy will require further validation in infection models.

### 3.5 The Modified Crone's model can be used to screen the antimicrobial potency of new drugs of interest

Besides ranking the relative potencies of known antimicrobials, the MCM can be applied to screen new compounds with suspected antibiofilm activity. To this end, a 4-day *in vitro*-matured biofilm is applied, which is exposed to the test compounds, without replenishing nutrients during the treatment phase. This nutrient-limited condition helps maintain bacteria in a quiescent state, which is representative of difficult-to-treat biofilms. Such parameters likely increase assay sensitivity (reducing false positives) but may reduce specificity (increasing the risk of false negatives).

Of the tested compounds, *cis*-2-decenoic acid (C2DA), diffusible signal factor (DSF), 3-oxo-dodecanoyl l-homoserine lactone (HSL), ebselen, and amylmetacresol significantly reduced *S. aureus* numbers in combination with clindamycin relative to the untreated control group (Fig. 4). Furthermore, C2DA, DSF, and amylmetacresol, in combination with clindamycin, were able to completely eradicate the approximately 10^8^ CFU *S. aureus* in the MCM (Fig. 4). However, it should be noted that clindamycin + HSL and clindamycin + Ebs were not significantly different from clindamycin monotherapy (data not shown).

## 4 Discussion

The development of effective anti-biofilm therapeutics is hampered by the lack of simple, scalable, and physiologically relevant *in vitro* models for biofilm growth. Most traditional biofilm models rely on liquid-based systems or specialized platforms that fail to capture host tissues' structural and environmental constraints. In contrast, the MCM provides a semi-solid, soft-tissue-like matrix for embedded biofilm formation, offering a reproducible and accessible platform for screening antimicrobial activity under conditions more representative of *in vivo* infection sites.

Numerous *in vitro* models have been developed to study bacterial biofilms, each with distinct advantages and limitations reviewed extensively elsewhere [13,14,31]. For instance, colony biofilms grown on agar surfaces offer high reproducibility but lack the spatial confinement and oxygen limitations typical of tissue-associated infections. Similarly, microtiter plate models allow for high-throughput analyses but are often conducted under high-shear, high nutrient availability, and well-aerated conditions that are not reflective of many infection microenvironments [32]. Flow cell systems, bioreactors, and the Calgary biofilm device introduce greater complexity but often require custom-made equipment, intricate protocols, and growth in purely liquid media.

Custom growth media incorporating serum, plasma, or tissue extracts can improve physiological relevance [14], but introduce batch-to-batch variability and reduced reproducibility. In contrast, the MCM was developed using commonly available laboratory reagents to simulate low-oxygen, low-shear, semi-solid environments such as those found in soft tissues, bone, or implanted materials. Although it lacks the biological complexity of *in vivo* host factors, the MCM allows for consistent growth of biofilm aggregates under spatial and diffusional constraints that closely mimic clinical infection environments [2]. Other agar-based *in vitro* biofilm models do exist [33], such as Kucera et al.*’s* artificial wound bed, which matures biofilm in a liquid, wound exudate-mimicking media, inside a pipette tip that is then harvested and placed in a two-layer semisolid agar medium [34]. Other embedment models include the use of collagen in combination with complex wound-simulating media [35,36], bacteria-inoculated agar beads layered with alginate in culture medium [37], and alginate beads [38], for example.

While the MCM reproduces essential physical constraints of tissue-like environments, it remains a deliberately simplified model. The agar matrix likely creates oxygen and nutrient gradients that generate some degree of heterogeneity within biofilms, but host-derived elements such as proteins, immune cells, and dynamic mechanical forces are absent. These simplifications were intentional, as the inclusion of complex host components (e.g., blood, plasma, or immune cells) would introduce high variability between laboratories and reduce reproducibility. Thus, the MCM should be regarded as a reductionist system that captures structural confinement and diffusional limitations relevant to *in vivo* infections, while not replicating the full biological complexity of host-pathogen interactions. While MCM-grown aggregates more resembled *in vivo* biofilms in terms of size, our comparisons were based solely on morphological dimensions. We did not assess physiological characteristics such as metabolic activity, extracellular matrix production, or the presence of dormant versus active subpopulations. Likewise, although nutrient and oxygen gradients are likely to develop within the agar plug, these were not quantified. Thus, the designation of MCM biofilms as ‘in vivo-like’ is limited to their physical dimensions, and future studies are needed to determine whether they also reproduce key physiological features of infection-associated biofilms.

We showed that antibiotics establish a homogeneous distribution at the site of biofilm in the MCM in 14 h, using clindamycin as a proxy. Larger molecules are expected to distribute more slowly, and smaller molecules are expected to distribute relatively faster. Other factors that could influence the distribution time of the antimicrobials are the pH and the resulting electrical charge of the molecules and whether these microspecies interact with the agar polymers. Other than the charge and size of the antibiotic molecules, in this model, the concentration of antibiotics is given as μg/mL to be comparable to units utilized in clinics. While results were presented in μg/mL to align with typical clinical units, molar concentration may be more appropriate for compound ranking to account for molecular weight and reduce potential bias.

We observed that liquid-cultured biofilms were significantly larger and more variable than those grown in semi-solid media. This may be partially explained by greater access to nutrients and oxygen in well-mixed liquid environments and fewer physical constraints on biofilm expansion. In contrast, the spatial confinement and reduced diffusion in agar-embedded cultures should more closely reflect *in vivo* environments and may help stabilize biofilm size, oxygen/nutrient gradients, and subsequent metabolic heterogeneity, but this should be studied in future studies.

Biofilm volume shapes the internal microenvironment, affecting antimicrobial susceptibility [1,2,17]. Larger biofilms tend to establish steeper diffusion gradients, which create metabolically distinct zones and contribute to developing antibiotic-tolerant subpopulations, such as persister cells. These gradients also reduce drug penetration, further complicating treatment. While biofilm size is not the only factor in tolerance, it is strongly intertwined with the physiological state and spatial architecture that drive antimicrobial recalcitrance [[39], [40], [41], [42]].

Using the MCM, we identified key differences in antimicrobial efficacy rankings between semi-solid and liquid-phase biofilm cultures. The relative potency of tested antibiotics differed between the two systems in some cases, even though the growth medium composition and exposure times were comparable. Notably, the quorum-sensing-disrupting compound DSF had no activity against planktonic cells but exhibited potent activity against biofilm-embedded bacteria – effects not captured in standard liquid-phase assays. These results highlight the importance of selecting biofilm-relevant models when screening for anti-biofilm agents or evaluating the synergistic potential of compound combinations.

Antimicrobial efficacy screening using the MCM confirmed the anti-biofilm-potentiating effects of several compound combinations. Among the compounds tested, C2DA, DSF, and amylmetacresol – when combined with clindamycin – significantly reduced CFU counts compared to clindamycin alone. This suggests the model's utility in detecting synergistic or potentiating effects, especially in compounds that target biofilm-specific processes, such as quorum-sensing or metabolic reactivation.

In this work, the MCM was also used to screen the antimicrobial activity of various compounds. Pyruvate was investigated as it was thought to potentially “awaken” dormant cells (by increasing their proton motive force [[43], [44], [45]]) and, in combination with clindamycin, may lead to a higher kill fraction. Tannic acid [46] and azithromycin [47] were suspected to inhibit biofilm formation, which may lead to enhanced susceptibility towards antimicrobials. 3-oxo-dodecanoyl-l-homoserine lactone (HSL) was previously shown to interfere with quorum sensing in *S. aureus* [48] and likewise for hamamelitannin, which was also reported to increase the susceptibility of *S. aureus* toward antimicrobials [49]. Ebselen, an organoselenium compound, has previously been shown to reduce established *S. aureus* biofilm [[50], [51], [52]]. Antitussives and mucolytics from cold medicines were also tested with the hypothesis that they might generally be able to disperse bio-hydrogels, such as biofilm. These were ambroxol, bromhexine, guaifenesin, and amylmetacresol. Furthermore, certain unsaturated fatty acids have been reported to induce dispersion of biofilms [[28], [29], [30],[53], [54], [55], [56], [57]], which were investigated alone and in combination with clindamycin. Of all the tested drug combinations, only C2DA, DSF, and amylmetacresol, in combination with clindamycin, significantly reduced CFU numbers when compared to clindamycin monotherapy.

Lastly, we studied whether antibiotic recalcitrance differs depending on whether biofilm developed in semi-solid conditions or whether they are associated with bone tissue or implant surfaces. We showed that the total CFU after 4 days of incubation, whether biofilms were agar-embedded, bone-associated, or implant-associated, was statistically indistinguishable. Likewise, the antimicrobial effect of levofloxacin in reducing CFUs after 3 days of drug incubation was statistically indistinguishable despite the different microenvironments for biofilm development. However, there may still be differences if other classes of antibiotics are investigated by applying different bacterial species or strains or in a setting with more complex host interactions, such as immune cells. The difficulties in clearing implant-associated biofilms may, instead of biofilm recalcitrance, reflect the challenge of achieving sufficient antibiotic concentrations at the site of infection *in vivo*.

This study focused on *S. aureus* as a model organism due to its clinical relevance and well-established biofilm-forming ability. While biofilm behaviour can vary between species, the MCM was designed to simulate general *in vivo*-like conditions, such as spatial confinement, limited diffusion, and low shear stress, that should apply broadly across bacterial taxa. These physical and chemical constraints are expected to influence other species similarly, promoting physiologically relevant biofilm development.

However, species-specific differences in biofilm structure and susceptibility may still occur. Future validation using additional pathogens will be important to confirm the model's broader applicability. Also, chronic infections are frequently polymicrobial, and interspecies interactions can significantly influence biofilm architecture, tolerance, and antimicrobial susceptibility. Although the present study focused on *S. aureus*, the MCM could readily be adapted to multispecies communities by co-inoculating different bacterial strains, or by varying the timing and ratio of their introduction. Such flexibility would allow investigation of how interspecies dynamics affect drug efficacy in a controlled, semi-solid environment. Future studies should explore this adaptation to broaden the applicability of the MCM for modeling complex, clinically relevant biofilms.

In conclusion, the MCM represents a simple and reproducible biofilm model suitable for evaluating antimicrobial potency under semi-solid growth conditions. Its design may offer a more physiologically relevant alternative to liquid-based biofilm assays by reproducing key physical constraints such as spatial confinement and diffusional limitation. Although the model is intentionally reductionist and does not capture the full biological complexity of host environments, this simplicity ensures reproducibility and accessibility across laboratories. As such, the MCM is well suited for preclinical screening of novel or repurposed compounds, and with further validation, could support the development of standardized protocols for biofilm susceptibility testing.

## CRediT authorship contribution statement

**Albert Fuglsang-Madsen:** Writing – review & editing, Writing – original draft, Visualization, Validation, Software, Project administration, Methodology, Investigation, Formal analysis, Data curation, Conceptualization. **Lasse Andersson Kvich:** Writing – review & editing, Writing – original draft, Validation, Methodology, Investigation. **Nicole Lind Henriksen:** Writing – review & editing, Writing – original draft, Validation, Project administration, Investigation. **Rasmus Kristensen:** Investigation. **Jonas Rosager Henriksen:** Supervision, Resources, Project administration, Funding acquisition, Conceptualization. **Anders Elias Hansen:** Supervision, Resources, Project administration, Funding acquisition, Conceptualization. **Thomas Bjarnsholt:** Writing – review & editing, Supervision, Resources, Methodology, Funding acquisition, Conceptualization. **Tim Holm Jakobsen:** Writing – review & editing, Writing – original draft, Supervision.

## Funding

This work was funded by the 10.13039/501100009708Novo Nordisk Foundation (Distinguished Innovator, grant number 0080941), the Innovation Fund Denmark (Innoexplorer, grant number 1048-00027B) and the Novo Nordisk Foundtion (Challenge, grant number NNF19OC0056411).

## Declaration of competing interest

The authors declare the following financial interests/personal relationships which may be considered as potential competing interests: Anders Elias Hansen reports financial support was provided by Novo Nordisk Foundation, Denmark. Anders Elias Hansen reports financial support was provided by Innovation Fund Denmark. If there are other authors, they declare that they have no known competing financial interests or personal relationships that could have appeared to influence the work reported in this paper.

## Data Availability

Data will be made available on request.
