# Supplementary material for: A semi-solid in vitro biofilm model for evaluating antimicrobial potency and biofilm-specific activity
Source: Biofilm. 2025 Nov 5;10:100328. doi: 10.1016/j.bioflm.2025.100328 (PMC12670108; doi:10.1016/j.bioflm.2025.100328)
Supplement: Multimedia component 1 [file mmc1.docx]

# Supplementary Material

**Supplementary Methods**

Detailed description and protocol to construct the Modified Crone’s Model

The MCM is constructed as follows:

- Make an overnight culture of a *S. aureus* strain of interest
- Insert two autoclaved metal grinding balls in each autoclaved test tube
  - Include three tubes per experiment as controls
- Add 150 µL of 0.5 % agar LB broth to each test tube. Let solidify
- Add 20 µL of 0.1 OD_600_ diluted overnight culture of *S. aureus*. Let the droplet dry
  - If comparing different strains, inoculate with a pre-determined number of colony-forming units (CFUs)
- Add 50 µL of liquid, lukewarm 0.5 % agar LB broth. Let solidify
- Add another 100 µL of liquid, lukewarm 0.5 % agar LB broth. Let solidify
- Add 600 µL of liquid LB broth on top
- Incubate at 37 °C/0 rpm for 96 hrs to mature biofilm.
- Inject 100 µL vehicle control (same as solvent) or 0.5 mg drug of interest (DOI) in 100 µL solvent (5 mg/ml). Do not add additional growth media, which may otherwise interfere with slow-growing bacterial subpopulations. When two APIs were used, the concentrations were doubled (10 mg/ml) and the added amount was halved (50 µl), ensuring a final concentration of each API of 0.5 mg/ml.
  - Initially, a final concentration of 0.5 mg/ml linezolid was chosen for the study, based on nine prior studies which measured *in situ* linezolid concentrations in soft tissue infections such as subcutaneous tissues, skeletal muscles and cancellous bone [1–9]. From their findings across both healthy- and infected individuals, linezolid concentrations ranged from 6.6 µg/ml to 17.4 µg/ml, which were achieved following administration of 600 mg intravenous injections or per oral. However, the concentration of other antibiotics *in situ* ranges from <1 µg/ml (e.g., azithromycin, flucloxacillin, colistin) up to 250-500 µg/mL (fosfomycin, imipenem) [10]. Thus, to take a range of antibiotics, and potential variations at body sites into account, 500 µg/ml was chosen as a conservative concentration, which was kept identical for subsequently tested DOIs, to maintain comparability.
  - Solvents will typically be dimethyl sulfoxide (DMSO), ethanol, phosphate-buffered saline (PBS), or sterile, demineralized water.
- Incubate for 72 hrs at 37 °C/0 rpm to allow DOI concentration equilibrium.
- Resuspend the liquid phase and discard it. Add 1 mL sterile saline at 4 °C
- Homogenize the content of each tube in a ball mill for 20 minutes (1500 rpm) at room temperature
- Centrifuge at 5,000 xg for 10 minutes/5 °C to pellet bacteria
- Discard supernatant (removal of residual antibiotics)
- Resuspend pellet in 700 µL sterile saline at 4 °C. Vortex tubes briefly, and sonicate at 4 °C for 2x5 minutes to disperse biofilm aggregates (e.g., on a Branson 1510 ultrasonic cleaner).
- Make a dilution series of each test tube and plate 30 µL droplets on 1.5 % agar LB plates (or Mannitol salt agar (MSA) plates, for *S. aureus* selection). Let dry and incubate bottom-up for 18-24 hrs at 37 °C/0 rpm
  - Also, save the undiluted samples at 4 °C. If no colonies are detected, the remaining content will be necessary.
- Count CFUs and calculate undiluted CFU numbers (see Supplementary Equation 1). If no colonies are observed in the undiluted sample, plate the entire test volume on a separate agar plate to confirm biofilm eradication (37 °C incubation for 48 hrs). This means the entire test volume was not added LB medium and cultured with shaking for 48 hrs at 37 °C in liquid media, to confirm eradication in that manner. That could be an alternative confirmatory criterion.
  1. If colonies are observed at lower dilutions but not in the higher dilutions, repeat the resuspension-centrifugation step in the undiluted sample to remove residual antibiotics further. Then repeat the plating step.

**Supplementary Equation 1**. Calculation of logarithmic transformed, undiluted, CFU numbers

$${log(n}_{CFU})={log}_{10}\left( \frac{n_{counted}}{V_{droplet}}\cdot V_{sample}\cdot d_{fac}+1 \right)$$

Where log(n_CFU_) is the logarithmic total number of CFUs in the undiluted sample, n_counted_ is the number of CFUs counted after culturing, V_droplet_ is the volume spot-plated on the agar plate (30 µL; see step 15), V_sample_ is the total volume of the spot-plated sample, and d_fac_ is the dilution factor. Note that if the sample volume is 1000 µL and 30 µL is spot-plated, the detection limit is ≈ 33 CFU/mL. For the purpose of logarithmic transformation, +1 was added to each value.

*Pseudomonas aeruginosa* biofilm cultured in the MCM

With the purpose of comparing the MCM model and liquid culturing also for Gram-negative bacteria, a laboratory *Pseudomonas aeruginosa* strain PAO1 (expressing superfolder GFP) was applied in standard a flow chamber assay (described elsewhere, e.g., [11]) and cultured for 2-3 days (24-72 hrs). Conversely, PAO1 was applied in the MCM as described for *S. aureus* in this study (Supplementary Figure 2). Image analysis was conducted as mentioned in the *Image Analysis* section of Materials & Methods. A Mann-Whitney nonparametric t-test was carried out in Prism 10 to statistically test differences in aggregate sizes. For liquid cultures, five ZStacks were analysed; three after 48 hrs of culturing and two after 72 hrs of culturing. One of the 48 hrs- and one of the 72 hrs belong stem from a separate experimental replicate, whereas the remaining two- and one of the 48- and 72 hrs, respectively, belong to another experimental replicate, each conducted at different times. For MCM-grown biofilm, two ZStacks were analysed, both originating from one experiment, but from two individual biological replicas.

Antibiotic diffusion assay

An experimental diffusion experiment was carried out to test the time for which antimicrobials distribute homogeneously throughout the MCM. Three tubes were prepared as described in “Construction of the biofilm model”; however, they were not inoculated by bacteria. Additionally, three tubes in which the 0.5 %-agar LB media was replaced by 1.5 %-agar LB, and three tubes were prepared, each containing 300 mg trabecular bone tissue from a healthy pig, which was centrifuged at 5,000 xg for 10 minutes to form a plug. 600 µL of liquid LB media was added to all tubes on top of the agar/tissue plug. The bottom of each tube was cut off, 100 µL clindamycin was injected (0.5 mg), and each tube was closed and placed in a well of a 12-well plate containing 2 mL PBS. Tubes were secured with tape in an upright position and kept in a highly humid plastic box to avoid evaporation at 37 °C. At specified time points (3 hrs, 1 day, 2-, 3-, and 7 days), 1 mL aliquots were retrieved from the sink media, and PBS was replenished. To extract clindamycin from the PBS aliquots, which also contained residues of LB media, samples of aliquots were mixed 1:5 with acetonitrile (MeCN), vortexed, and centrifuged at 15,000 xg for 15 mins/23 °C. The extraction method was validated (Supplementary Figure 1).

In the MCM, injected antibiotics slowly diffuse through the agar to establish an equilibrium of homogeneous concentration. Exponential plateau models were applied to fit the data, as shown in Supplementary Equation 2. The disc comprising *S. aureus* is located at a depth of 0.15 cm^3,^ i.e., halfway through the agar, with a total volume of 0.3 cm^3^. The diffusion rate (Supplementary Tables 1 and 2) determined the time it takes for a homogeneous concentration of antibiotics to establish in the 0.5 % agar-LB model, as described in Supplementary Equation 2.

**Supplementary Equation 2.** General equation for exponential plateau models used in diffusion experiments.

$$y=y_{max}-\left( y_{max}-y_{0} \right)\cdot e^{-k\cdot t}$$

Where y_max_ is the total amount of clindamycin (measured in µg), y_0_ is the starting amount (in µg) and k is the rate constant. Y is the amount of clindamycin (in µg) diffused through the 0.3 cm^3^ agar plug at time t (in hrs).

HPLC analyses

Post-extraction samples were injected into an XBridge® BEH C18 column (2.5 µm, 4.6x75 mm at 25 °C) at a 1 ml/min flow rate for HPLC analysis. Solvent A comprised 5 % MeCN and 0.1 % v/v trifluoroacetic acid (TFA) (aq.), whereas solvent B comprised 99.9 % MeCN with 0.1 % v/v TFA. The gradient was as follows: 0 % solvent B for 1 min, 0 to 100 % solvent B for 5 mins, 100 % solvent B for 1.5 mins, 100-0 % solvent B over 0.5 mins followed by 0 % solvent B for 1 min. Clindamycin ultraviolet (UV) absorbance was detected at 3.5 mins at 202 nm. The HPLC system was a Nexera X2, and chromatograms were analyzed using Shimadzu LabSolutions Postrun Analysis software.

Minimum inhibitory concentration assay

For the purpose of comparing the potencies of known antimicrobials, MIC, MBEC and MCM assays were carried out. For the MIC determination, in each well of a 96-well plate, 150 μL of LB media was added alongside serial dilutions of linezolid, levofloxacin, clindamycin, rifampicin, cis-2-decenoic acid (C2DA) or cis-11-methyl-2-dodecenoic acid (diffusible signal factor, DSF), respectively, ranging from 0-8 μg/mL. Then, 10 μL of an overnight culture of *S. aureus* strain S54F9 was added to each well, diluted to OD_600_ = 0.1. After 24 hrs, the OD_600_ was measured. Each antimicrobial concentration was run in triplicate. The experiment was repeated for clindamycin and levofloxacin. The MIC was defined as the lowest concentration (in μg/mL) where there was no statistically significant difference from the untreated, non-inoculated control media.

Minimum biofilm eradication (MBEC) assay

Fifteen μL of *S. aureus* S54F9 overnight culture at OD_600_ = 1 were inoculated in the wells of a 96-well plate to a final volume of 150 μL of LB media/well (final OD_600_ = 0.01). The plate was given a peg-lid and was incubated at 37 °C for 1 day at 110 rpm. After incubation, the peg-lid was moved to two consecutive plates containing sterile PBS and swirled gently to detach non-biofilm adhered bacteria. The peg-lid was then moved into a new plate, where each well contained 150 μL of liquid LB media with serial dilutions of linezolid, levofloxacin, clindamycin, C2DA, DSF, or rifampicin and incubated for 1 day at 37 °C and 110 rpm. Inoculated wells without antibiotics and non-inoculated wells containing only growth media were used as controls. Following incubation, the peg-lid was washed twice in PBS before being transferred to a new LB-containing plate, and the plate was sonicated for 30 minutes at room temperature to dislodge biofilm, followed by incubation at 37 °C/110 rpm overnight. The following day, OD_600_ was measured, and MBEC was defined as the concentration at which there was no significant difference between the OD_600_ of the non-inoculated wells and the test wells.

**Supplementary Figures**


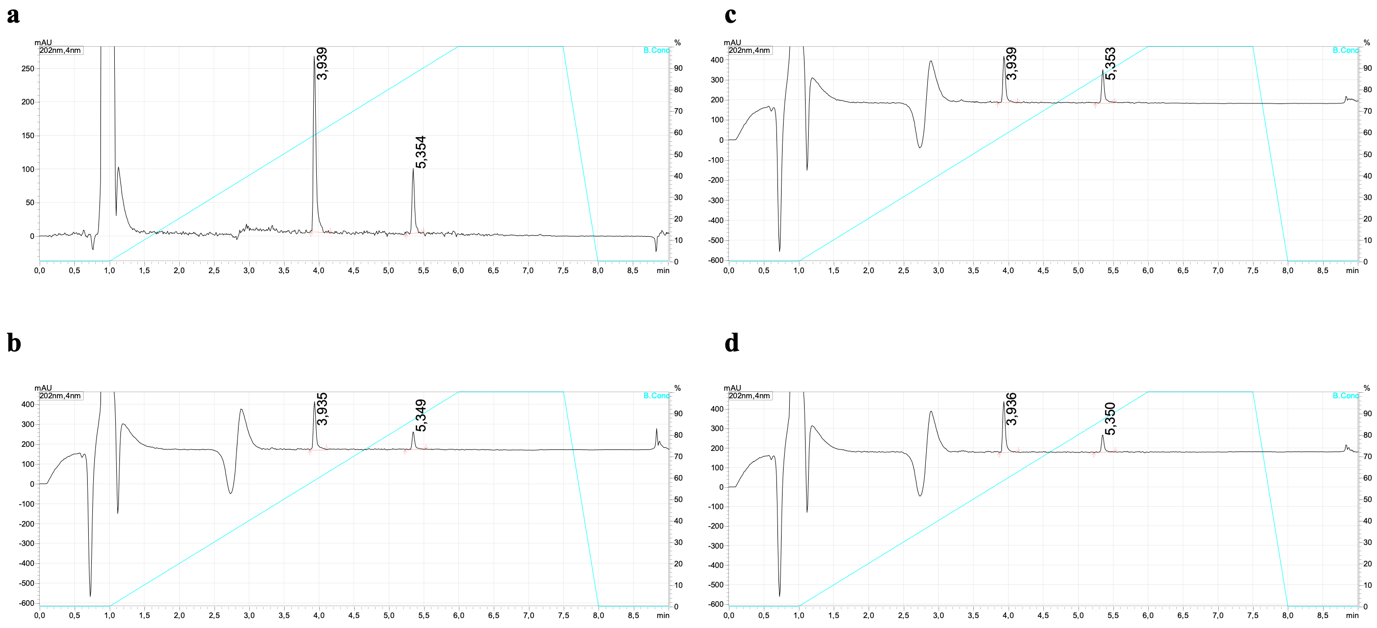


**Supplementary Figure 1.** Validation of a clindamycin extraction method from the LB-comprising sink media following diffusion through the agar plug of the modified Crone’s model. The chromatograms show the absorption of clindamycin (retention time at 3.9 minutes) and a compound from the LB media (retention time at 5.4 minutes) measured at 202 nm. More details can be found in Supplementary Methods. a) The chromatogram from a sample of 0.5 mg/mL clindamycin spiked subsequent to the extraction protocol. b-d) Chromatograms from three replicas of 0.5 mg/mL clindamycin (in diluted LB media), which underwent the extraction protocol. The measured AUCs of the control (post-extraction spike) was 726654. The AUCs of clindamycin in chromatograms b-d were 659020, 594237, and 693620. The extraction efficiency was 89.3 ± 4.0 %.


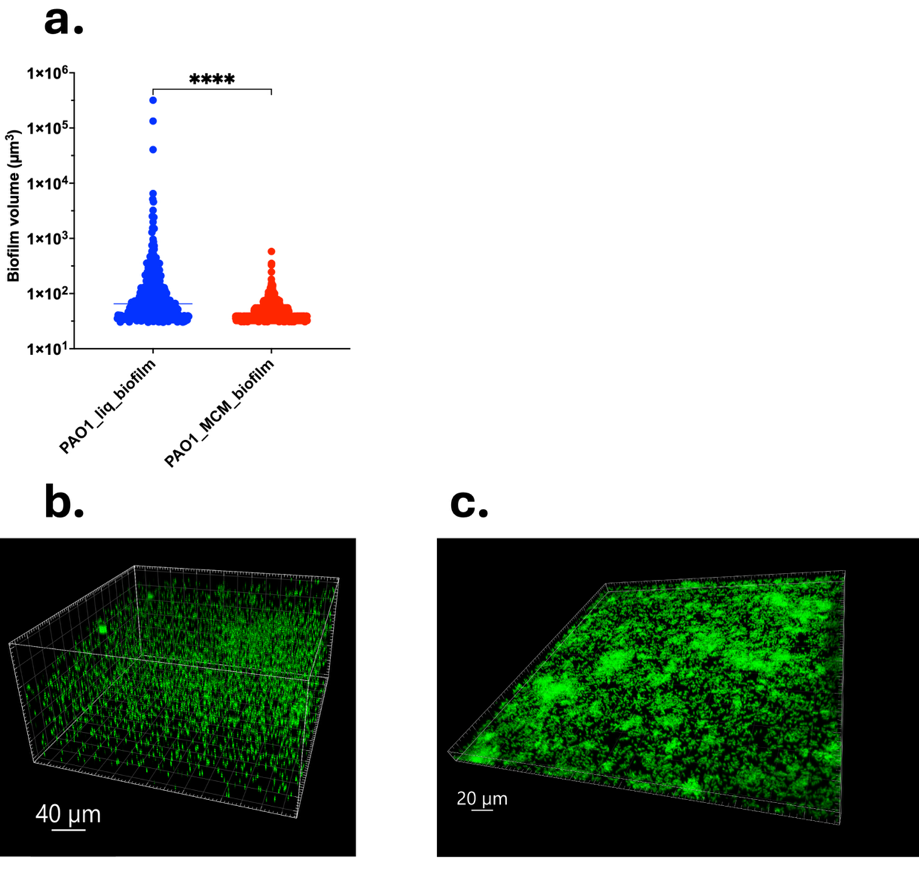


**Supplementary Figure 2.** Comparison of aggregate sizes from PAO1 cultures. A. shows the size distribution of aggregates in the two biofilm models (n_liq_ = 384, n_MCM._ 969). B. shows a ZStack from PAO1 cultured in the MCM. C. Shows PAO1 cultured in a classical flow chamber [11].


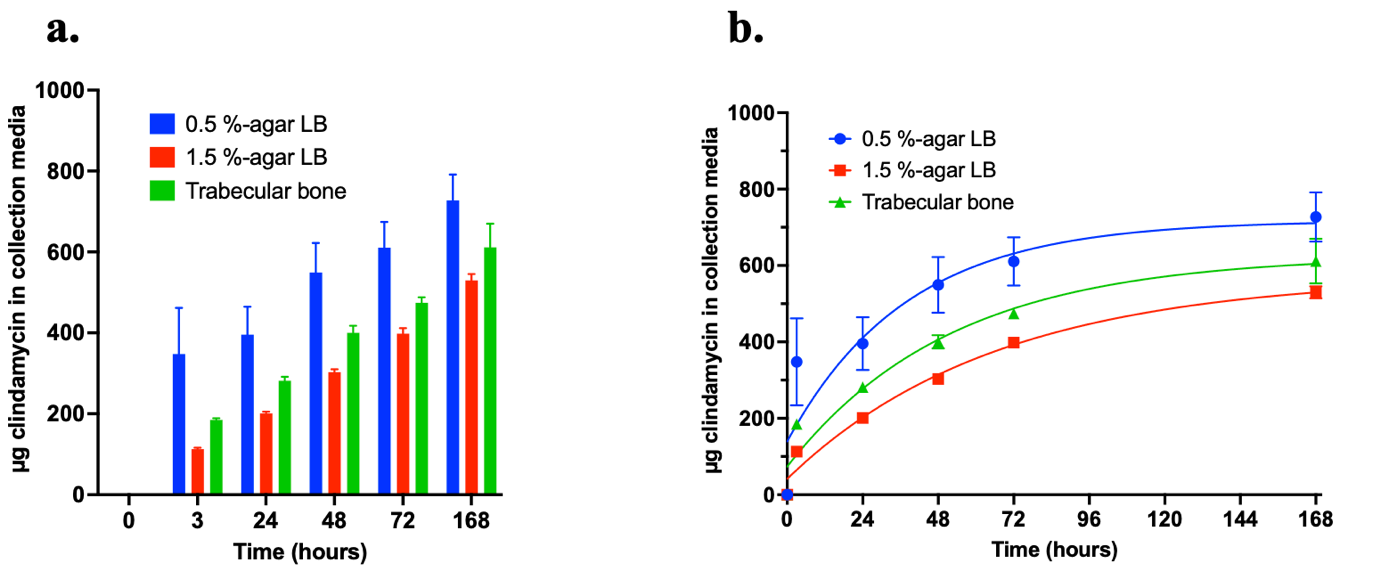


**Supplementary Figure 3.** Diffusion of clindamycin through the agar plug model. a) Graph showing the accumulated quantity of clindamycin having traveled through the agar or bone tissue plug over time. b) Progression of clindamycin (in µg) through the 0.3 cm^3^ agar- or trabecular bone tissue plug (functions can be found in Supplementary Tables 1 and 2). Three biological replicas were included in the experiment for each model (i.e., three measurements at each time point, for each model).

**Supplementary Table 1.** Exponential plateau fits in the clindamycin diffusion assay (i.e., experimentally determined), listed according to the general correlation: $y=y_{max}-\left( y_{max}-y_{0} \right)\cdot e^{-k\cdot x}$; see Supplementary Equation 2.

| Model variant | Exponential plateau function |
| --- | --- |
| 0.5 % agar-LB | $y=717.8-\left( 717.8-139.5 \right)\cdot e^{-0.026\cdot t}$ |
| 1.5 % agar-LB | $y=572.1-\left( 572.1-41.6 \right)\cdot e^{-0.015\cdot t}$ |
| Trabecular bone tissue | $y=626.6-\left( 626.6-73.6 \right)\cdot e^{-0.019\cdot t}$ |

**Supplementary Table 2.** Experimentally determined parameters from the exponential plateau fit (listed in Table 1) the clindamycin diffusion assay (see Supplementary Figure 2).

| Model variant | No. of replicas | R^2^ | y_max_ (µg) | y_0_ (µg) | k (rate constant) | 95 %-confidence interval of k |
| --- | --- | --- | --- | --- | --- | --- |
| 0.5 % agar-LB | 3 | 0.72 | 717.8 | 139.5 | 0.026 | 0.006718 to 0.7869 |
|  |  |  |  |  |  |  |
|  |  |  |  |  |  |  |
| 1.5 % agar-LB | 3 | 0.97 | 572.1 | 41.6 | 0.015 | 0.01072 to 0.02008 |
|  |  |  |  |  |  |  |
| Trabecular bone tissue | 3 | 0.92 | 626.6 | 73.6 | 0.019 | 0.01070 to 0.03200 |

**Supplementary Equation 3**. Construction of a general function quantifying the µg of clindamycin accumulating at the disc (i.e., at 0.15 cm^3^ agar, or half of the total agar volume) over time, t, given in hours. The rate constant used for the construction of the function was the experimentally determined rate constant listed in Supplementary Table 2.

$$y_{disc}=\frac{1}{2}\cdot\left( 500-\left( 500-0 \right)\cdot e^{-0.026\cdot t} \right)$$

**Supplementary Calculation 1.** Using **Supplementary Equation 3** to determine the time-to-equilibrium of 0.5 mg/mL (i.e., y = 75 µg) halfway through the agar plug (i.e., at a depth or volume of 0.15 cm^3^). The calculation assumes that the time to establish a homogeneous concentration in the upper liquid phase is near instantaneous, meaning that the diffusion rate is solely through the agar, and with the consideration that a concentration of 0.5 mg/mL corresponds to 75 µg in 0.15 cm^3^ (or 0.15 mL).

$$y_{disc}=\frac{1}{2}\cdot\left( 500-\left( 500-0 \right)\cdot e^{-0.026\cdot t} \right)\leftrightarrow$$

$$t=\frac{ln(\frac{-0.5\cdot(500-0)}{y-0.5\cdot500}}{0.026}\to y=75 \mu g \to\underline{t=13.72 hrs}$$

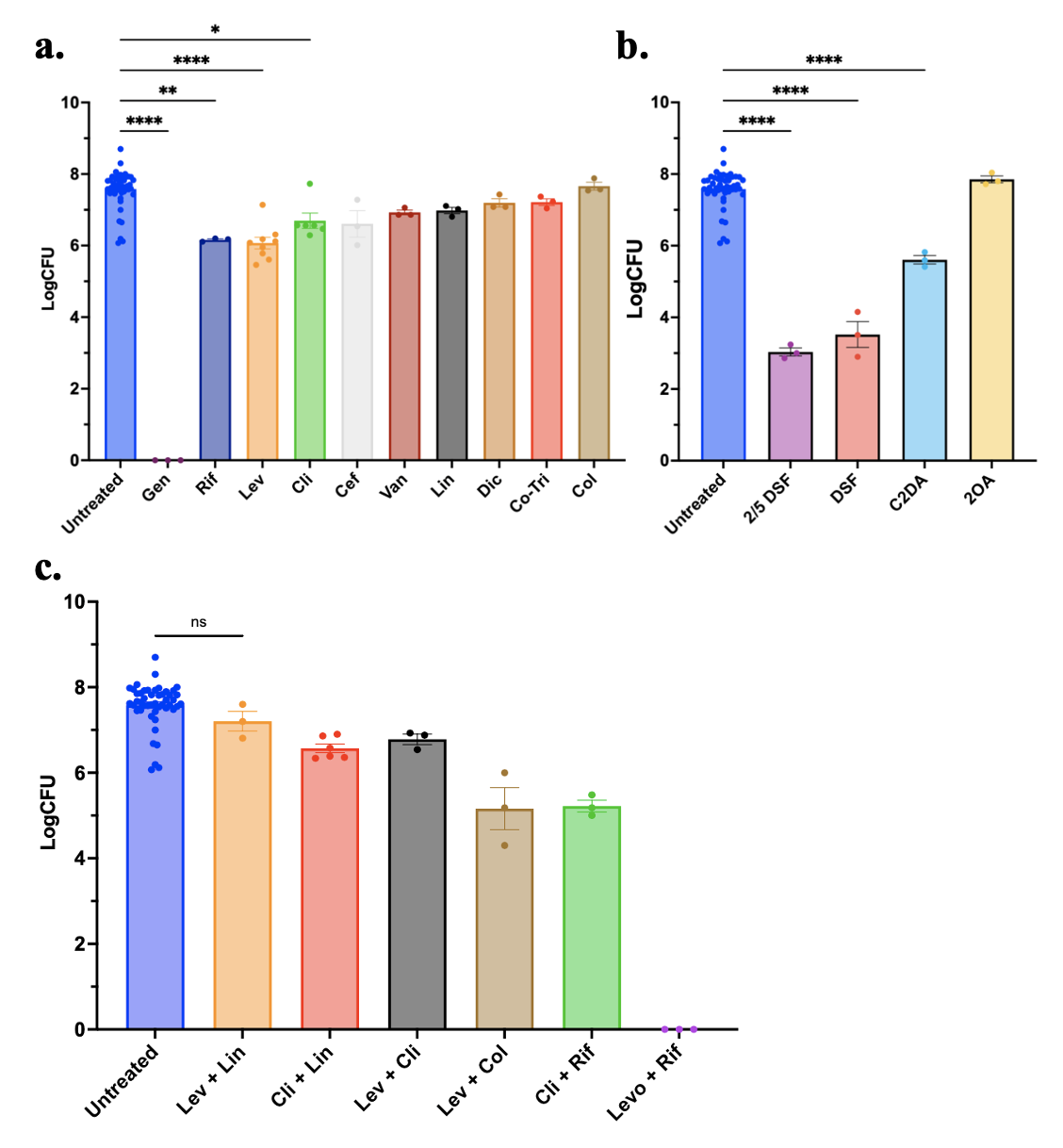


**Supplementary Figure 4. The antimicrobial effect of several compounds on *S. aureus* S54F9.** Four-day-old biofilm, exposed to each compound for 3 days: Gen = gentamicin. Rif = rifampicin. Lev = levofloxacin. Cli = clindamycin. Cef = cefalotin. Van = vancomycin. Lin = linezolid. Dic = Dicloxacillin. Co-Tri = co-trimoxazole. Col = colistin (negative control). DSF = diffusive signal factor (cis-11-methyl-2-dodecenoic acid). C2DA = cis-2-decenoic acid. 2OA = 2-octenoic acid. a) depicts typical antibiotics used in clinics for different bacterial infections. b) depicts unsaturated fatty acids previously shown to harbor antimicrobial activity. *: p ≤ 0.05. **: p ≤ 0.01. ****: p ≤ 0.0001. The number of biological replicas in each treatment group was 3, except for Clindamycin, where 6 biological replicas were conducted; three replicas in two independent experiments. Likewise, levofloxacin has 9 biological replicas: three replicas in three independent experiments. The untreated group in panels A and B are identical and comprise the 51 untreated samples across all seventeen MCM experiments.


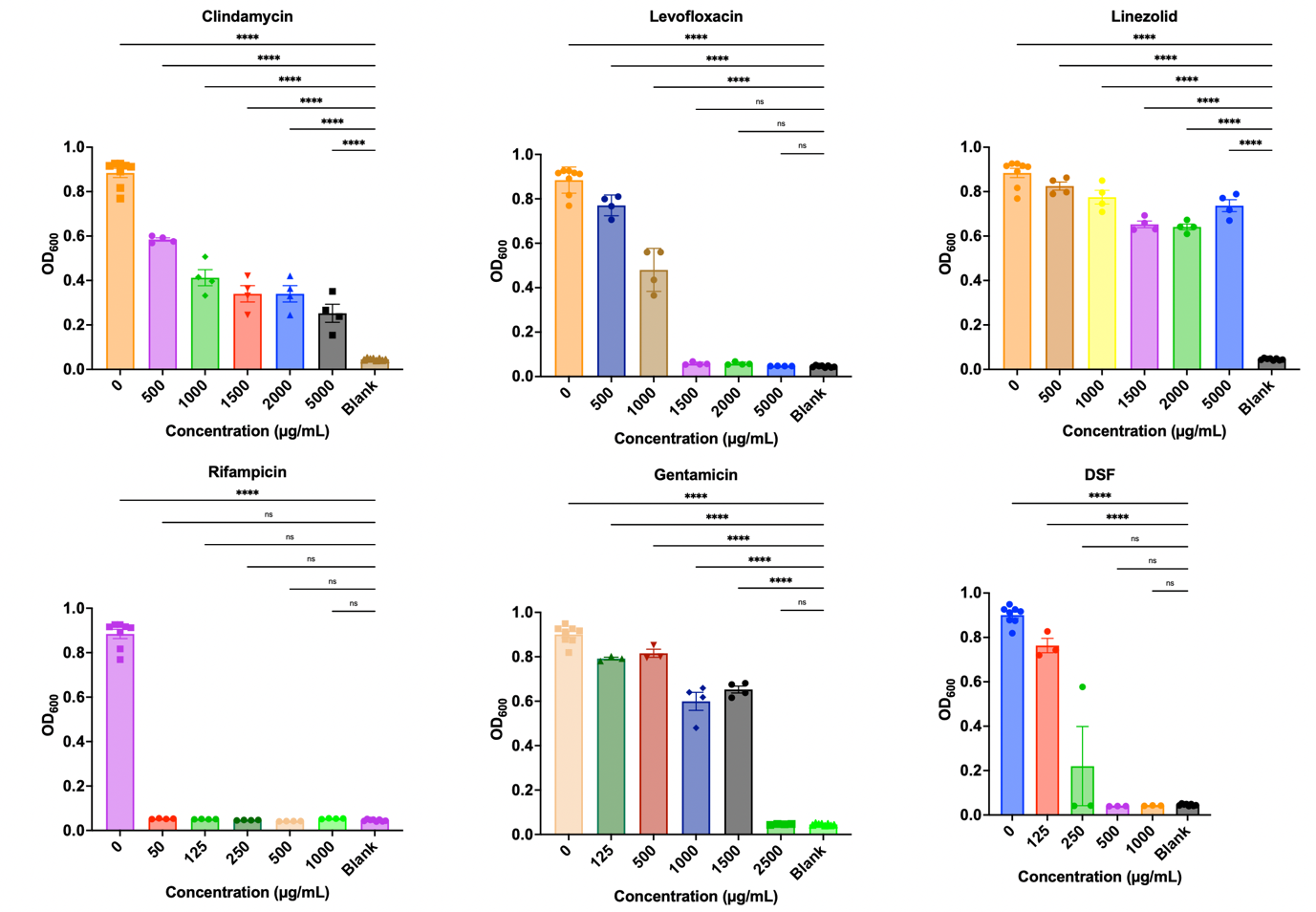


**Supplementary Figure 5.** MBEC determinations of levofloxacin, clindamycin, DSF (diffusible signal factor; cis-11-methyl-2-dodecenoic acid), linezolid, gentamicin, or rifampicin against *S. aureus* strain S54F9. MBECs were defined as the lowest concentration (in µg/mL) at which regrowth of bacteria was observed after their 24 h-old biofilm state had incubated with antimicrobials at given concentrations for 24 hrs. For the comparisons in Table 1, clindamycin and linezolid were ranked according to effect size at the highest tested concentration. Statistical data analyses were performed as multiple one-way ANOVA comparisons with correction (see Materials & Methods). For each concentration, 4 wells were used (4 technical replicas), except for the control, “0 µg/mL”, which comprised 9 wells. The control, or “0 µg/mL” is the same for clindamycin, levofloxacin, linezolid and rifampicin, as they were analysed in the same plate. Likewise, the 9 wells in gentamicin and DSF are identical as they were analysed in the same plate.


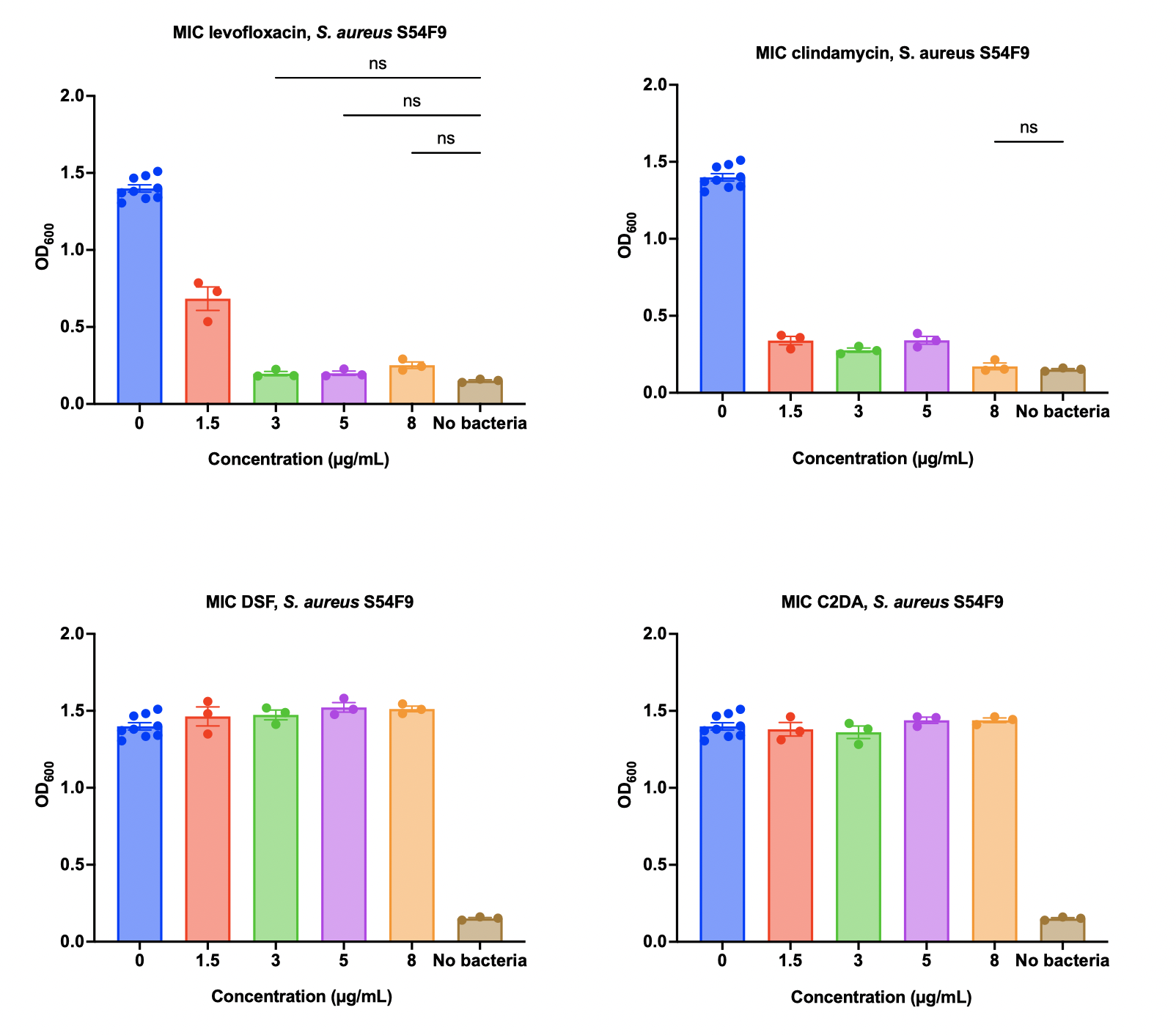

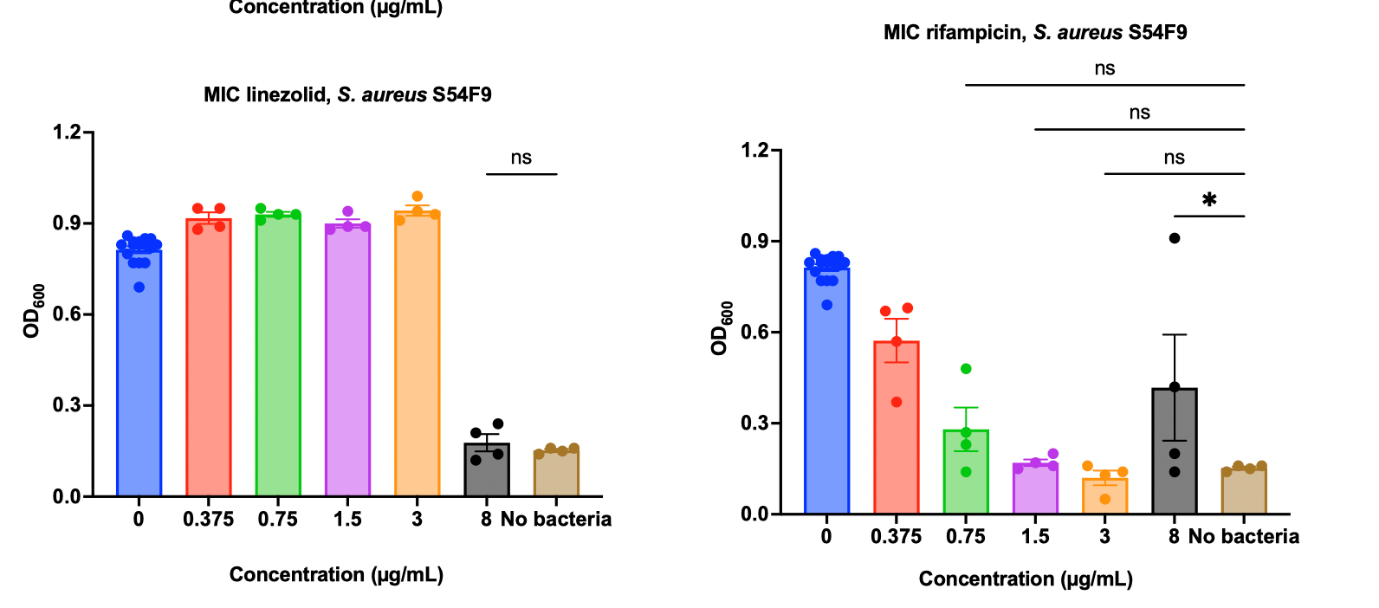


**Supplementary Figure 6.** MIC determinations of levofloxacin, clindamycin, DSF (diffusible signal factor; cis-11-methyl-2-dodecenoic acid), C2DA (cis-2-decenoic acid), linezolid, or rifampicin. MICs were defined as the lowest concentration (in µg/mL) at which bacterial growth was inhibited and thus no significant difference from growth media alone (“No bacteria”). Statistical data analyses were performed as one-way ANOVA multiple comparisons with correction by Tukey’s test (see Materials & Methods). The MIC of gentamicin was determined as 0.25 µg/mL in a previous study [12]. For MIC determinations of levofloxacin, clindamycin, DSF and C2DA, each concentration comprised 3 wells (i.e., 3 technical replicas), whilst their control “0 µg/mL” is identical and comprised 9 wells. The “no bacteria” control was also identical and comprised 3 wells. For MIC determinations of linezolid and rifampicin, each tested concentration comprised 4 wells (4 technical replicas) whilst the 0 µg/mL and “no bacteria” control were identical and comprised 16- and 4 wells, respectively.


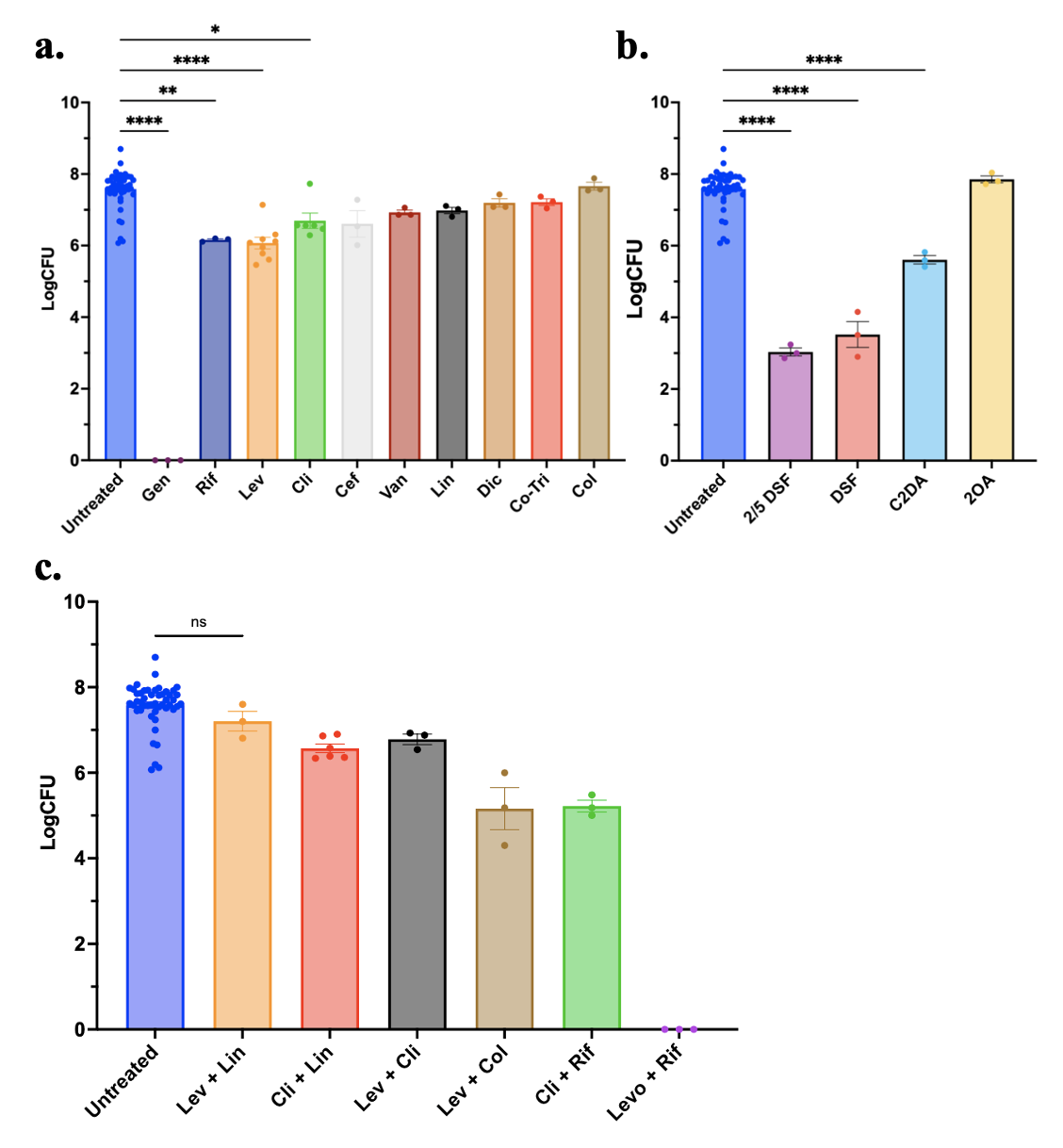


**Supplementary Figure 7.** Antibiotics were tested in combinations against a 4-day-*in-vitro*-matured biofilm (the modified Crone’s model). All combinations (except for levofloxacin + linezolid) statistically reduced the bacterial numbers relative to no treatment (based on a one-way ANOVA with correction for multiple comparisons; see Materials & Methods). No difference was found between clindamycin + linezolid relative to clindamycin alone or levofloxacin + colistin relative to levofloxacin alone. Furthermore, levofloxacin + clindamycin was significantly less antimicrobial than levofloxacin alone.

**Supplementary References**

1. Schwameis R, Syré S, Sarahrudi K, et al. Penetration of linezolid into synovial fluid and muscle tissue after elective arthroscopy. Journal of Antimicrobial Chemotherapy **2017**; 72:2817–2822.

2. Andreas M, Zeitlinger M, Wisser W, et al. Cefazolin and linezolid penetration into sternal cancellous bone during coronary artery bypass grafting. Eur J Cardiothorac Surg **2015**; 48:758–764.

3. Eslam RB, Burian A, Vila G, et al. Target site pharmacokinetics of linezolid after single and multiple doses in diabetic patients with soft tissue infection. The Journal of Clinical Pharma **2014**; 54:1058–1062.

4. Wiskirchen DE, Shepard A, Kuti JL, Nicolau DP. Determination of Tissue Penetration and Pharmacokinetics of Linezolid in Patients with Diabetic Foot Infections Using *In Vivo* Microdialysis. Antimicrob Agents Chemother **2011**; 55:4170–4175.

5. Koomanachai P, Keel RA, Johnson-Arbor KK, Suecof LA, Nicolau DP, Kuti JL. Linezolid penetration into wound tissue of two diabetic patients before and after hyperbaric oxygen therapy. Undersea Hyperb Med **2011**; 38:11–6.

6. Traunmüller F, Schintler MV, Spendel S, et al. Linezolid concentrations in infected soft tissue and bone following repetitive doses in diabetic patients with bacterial foot infections. International Journal of Antimicrobial Agents **2010**; 36:84–86.

7. Stolle LB, Plock N, Joukhadar C, et al. Pharmacokinetics of linezolid in bone tissue investigated by in vivo microdialysis. Scandinavian Journal of Infectious Diseases **2008**; 40:24–29.

8. Islinger F, Dehghanyar P, Sauermann R, et al. The effect of food on plasma and tissue concentrations of linezolid after multiple doses. International Journal of Antimicrobial Agents **2006**; 27:108–112.

9. Dehghanyar P, Bürger C, Zeitlinger M, et al. Penetration of Linezolid into Soft Tissues of Healthy Volunteers after Single and Multiple Doses. Antimicrob Agents Chemother **2005**; 49:2367–2371.

10. Nielsen MBD, Jørgensen AR, Stilling M, Mikkelsen MKD, Jørgensen NP, Bue M. Dynamic distribution of systemically administered antibiotics in orthopeadically relevant target tissues and settings. APMIS **2024**; 132:992–1025.

11. Christensen BB, Sternberg C, Andersen JB, et al. [2] Molecular tools for study of biofilm physiology. In: Methods in Enzymology. Elsevier, 1999: 20–42. Available at: https://linkinghub.elsevier.com/retrieve/pii/S0076687999100041. Accessed 30 October 2025.

12. Jensen LK, Bjarnsholt T, Kragh KN, et al. *In Vivo* Gentamicin Susceptibility Test for Prevention of Bacterial Biofilms in Bone Tissue and on Implants. Antimicrob Agents Chemother **2019**; 63:e01889-18.
